# Supplementary material for: The Very Long COVID: Persistence of Symptoms after 12–18 Months from the Onset of Infection and Hospitalization
Source: J Clin Med. 2023 Feb 28;12(5):1915. doi: 10.3390/jcm12051915 (PMC10003916; doi:10.3390/jcm12051915)
Supplement: Supplementary file 1 [file jcm-12-01915-s001.zip › jcm-2208242-supplementary.pdf]

Supplementary material Table S1. Therapies and laboratory exams at the time of hospitalization.

Item	Mild N=19	Moderate N=68	Severe N=34	P
Therapy				
Beta-blockers	1 (5.3)	12 (17.6)	4 (11.8)	0.630
ACE-inhibitors	0 (0)	8 (11.8)	6 (17.6)	0.407
Sartans	1 (5.3)	6 (8.8)	1 (2.9)	0.682
Warfarin	0 (0)	2 (2.9)	0 (0)	0.741
DOAC	0 (0)	2 (2.9)	2 (5.9)	0.707
Antiplatelet agents	0 (0)	14 (20.6)	5 (14.7)	0.234
Calcium antagonists	2 (10.5)	9 (13.2)	5 (14.7)	0. 917
Statins	3 (15.8)	12 (17.6)	2 (5.9)	0.537
Laboratory exams (acute phase)				
Peak fibrinogen (mg/dL)	576 (172)	583 (144)	681 (139)	0.009
Peak D-dimer (µg/mL)	1.5 (1.8)	3.7 (9.1)	2.8 (5.3)	0.651
Peak PLT (x1,000 cells/µL)	293 (85)	320 (106)	361 (121)	0.120
Nadir PLT (x1,000 cells/µL)	209 (86)	186 (69)	164 (60)	0.134
Nadir antithrombin (%)	95 (11.8)	99 (15.5)	104 (16.9)	0.135

Data are mean (standard deviation), or number (%).
ACE: angiotensin-converting enzyme; DOAC: direct oral anticoagulant.

Supplementary material Table S2. Patient population (N=121) data and association with major physical symptoms persistence.

Item	Hazard ratio	95% C.I.	P
Gender male	1.49	(0.68-3.25)	0.321
Body mass index (kg/m^2)	0,99	(0.90-1.10)	0.944
Hospital stay (days)	1.02	(0.971-1.6)	0.508
Intensive care unit admission	0.15	(0.01-2.08)	0.157
Obesity	0.78	(0.22-2.72)	0.695
Arterial hypertension	5.33	(0.32-88)	0.243
Diabetes	0.84	(0.27-2.46)	0.757
Coronaropathy	0.61	(0.19-1.93)	0.399
Heart failure	1.19	(0.23-6.12)	0.837
Smoking habit	1.52	(0.82-2.82)	0.187
Atrial fibrillation	2.61	(0.44-15.6)	0.293
Active cancer previous 5 years	1.40	(0.23-8.5)	0.713
Chronic obstructive pulmonary disease	0.46	(0.45-4.45)	0.503
Chronic kidney failure	2.33	(0.20-27.6)	0.502
Previous cerebrovascular accident	0.72	(0.15-19.6)	0.660
Anxiety	0.45	(0.12-1.67)	0.235
Depression	1.09	(0.31-3.82)	0.894
Chronic liver failure	0.30	(0.02-3.76)	0.351
Therapy			
Beta-blockers	1.03	(0.33-3.22)	0.954
ACE-inhibitors	1.57	(0.39-6.39)	0.528
Sartans	0.74	(0.15-3.59)	0.706
Warfarin	0.02	(0-6.32)	0.084
Direct oral anticoagulants	0.23	(0.01-8.19)	0.422
Antiplatelet agents	1.56	(0.39-6.39)	0.528
Calcium antagonists	1.96	(0.53-7.20)	0.309
Statins	0.44	(0.14-1.41)	0.165

Data are mean (standard deviation), median (interquartile range) or number (%).
ACE: angiotensin-converting enzyme.

Supplementary material Table S3. Patient population (N=121) data and association with major neurological symptoms persistence.

Item	Hazard ratio	95% C.I.	P
Age class	1.08	(0.88-1.32)	0.483
Gender male	1.15	(0.87-1.52)	0.311
Body mass index (kg/m^2)	0,99	(0.99-1.03)	0.931
Hospital stay (days)	1.01	(0.971-1.6)	0.330
Obesity	0.69	(0.44-1.06)	0.093
Arterial hypertension	4.36	(0.48-78)	0.443
Diabetes	0.57	(0.17-1.46)	0.557
Coronaropathy	0.60	(0.18-1.90)	0.389
Heart failure	1.03	(0.67-1.61)	0.880
Smoking habit	1.02	(0.73-1.44)	0.891
Atrial fibrillation	1.36	(0.84-2.20)	0.216
Active cancer previous 5 years	1.48	(0.25-8.6)	0.613
Chronic obstructive pulmonary disease	1.05	(0.56-1.94)	0.888
Chronic kidney failure	1.18	(0.58-2.39)	0.651
Previous cerebrovascular accident	1.97	(0.71-5.44)	0.192
Chronic liver failure	0.54	(0.33-1.03)	0.096
Therapy			
Beta-blockers	1.72	(0.95-2.22)	0.106
ACE-inhibitors	0.92	(0.59-1.45)	0.730
Sartans	1.48	(0.85-2.59)	0.167
Warfarin	0.47	(0.23-1.02)	0.067
Direct oral anticoagulants	1.30	(0.61-2.73)	0.493
Antiplatelet agents	0.87	(0.58-1.31)	0.502
Calcium antagonists	0.72	(0.49-1.06)	0.101
Statins	1.61	(0.96-2.72)	0.074

Data are mean (standard deviation), median (interquartile range) or number (%).
ACE: angiotensin-converting enzyme.
